# Usability of Virtual Visits for the Routine Clinical Care of Trans Youth during the COVID-19 Pandemic: Youth and Caregiver Perspectives

**DOI:** 10.3390/ijerph182111321

**Published:** 2021-10-28

**Authors:** Carolina Silva, Alex Fung, Michael A. Irvine, Shabnam Ziabakhsh, Brenden E. Hursh

**Affiliations:** 1 Department of Pediatrics, Division of Endocrinology, British Columbia Children’s Hospital and University of British Columbia, 4480 Oak Street, Vancouver, BC V6H 3V4, Canada; carolina.silva@cw.bc.ca (C.S.); Alex.Fung@cw.bc.ca (A.F.); 2 Biostatistics Core, Clinical Research Support Unit, BC Children’s Research Institute, 938 W 28th Ave, Vancouver, BC V5Z 4H4, Canada; Mike.Irvine@bcchr.ca; 3 Women’s Health Research Institute, BC Women’s Hospital and Health Centre, 4500 Oak Street, Vancouver, BC V6H 3N1, Canada; SZiabakhsh@cw.bc.ca

**Keywords:** transgender, health services for transgender persons, adolescents, telemedicine, virtual, patient satisfaction, usability

## Abstract

We evaluated families’ perspectives on the usability of virtual visits for routine gender care for trans youth during the COVID-19 pandemic. An online survey, which included a validated telehealth usability questionnaire, was sent to families who had a virtual Gender Clinic visit between March and August 2020. A total of 87 participants completed the survey (28 trans youth, 59 caregivers). Overall, usability was rated highly, with mean scores between “quite a bit” and “completely” in all categories (usefulness, ease of use, interface and interaction quality, reliability, and satisfaction). Caregivers reported higher usability scores compared to trans youth [mean (SD) 3.43 (0.80) vs. 3.12 (0.93), *p* = 0.01]. All families felt that virtual visits provided for their healthcare needs. A total of 100% of youth and caregivers described virtual appointments as safer or as safe as in-person visits. A total of 94% of participants would like virtual visits after the pandemic; families would choose a mean of two virtual and one yearly in-person visit with a multidisciplinary team. Overall, virtual gender visits for trans youth had impressive usability. Participants perceived virtual visits to be safe. For the future, a combination of virtual and in-person multidisciplinary visits is the most desired model.

## 1. Introduction

“Transgender” is a broad term encompassing people whose gender identities are not aligned with their sex recorded at birth [1]. In this report, we use the term “trans youth” to include children, adolescents, and young adults with a variety of gender identities, including those who do not conform to conventional “male” or “female” roles, but rather identify between, outside and beyond the gender binary (non-binary or gender-diverse identities, among others) [2,3]. Gender dysphoria refers to the discomfort or distress caused by the discrepancy between a person’s gender identity and their sex assigned at birth [4]. The prevalence of self-reported transgender identity ranges between 1 to 9%, and may be even higher at younger ages, although the proportion of children and adolescents who experience gender dysphoria is not well known [5,6,7].

Gender-affirming care has shown significant benefits in terms of well-being and quality of life in this population [8,9,10,11]. Access to health care for youth who are transgender has been evolving over recent years. In the last two decades, the number of adolescents referred to gender clinics in Europe and North America has significantly increased [12,13,14]. Nevertheless, there are still many barriers to accessing gender-related care, including distance to larger urban areas where most specialized clinics are concentrated. A significant proportion of trans youth in Canada recently reported that they are not receiving the medical attention that they need [15,16,17].

While trans youth already face many health disparities, the COVID-19 pandemic has posed further challenges by making access to care even more difficult for this population [18,19]. For example, early in the pandemic, BC Children’s Hospital (BCCH) in British Columbia, Canada, canceled all in-person non-urgent outpatient medical visits. In this setting, there was a critical need for innovative solutions to provide gender-affirming treatment during this complex time.

Telemedicine, defined as the use of electronic information and communication technologies to provide and support health care when distance separates the participants [20], is an option to improve the delivery of care to trans youth. We use the term “virtual visit” to refer to a clinical encounter in which the patient and physician are communicating with video and audio using a phone, tablet, or computer. In other patient populations, similar visits have been shown to not only increase access to care but also to be feasible and acceptable [21]. In line with this, our team has found that youth with type 1 diabetes and their families were overall satisfied with virtual visits and considered these useful [22]. Even before the onset of the COVID-19 pandemic, a study showed that trans youth were interested in receiving telemedicine, particularly for ongoing care and monitoring [23]. There is now increasing evidence to support the feasibility of telemedicine in gender-affirming care to increase access to care [24,25,26,27]. In studies focused on experiences and attitudes, trans youth and families have had positive perceptions of virtual gender-related health care during the COVID-19 pandemic [28,29].

During the early stages of the current pandemic, the Gender Clinic at BCCH rapidly shifted from in-person to virtual care for all routine clinic visits. The objective of this study was to evaluate the usability of virtual visits for the gender-related care of trans youth during the COVID-19 pandemic. We also aimed to gain insight into the experience of participants in these visits and learn about their future preferences.

## 2. Materials and Methods

### 2.1. Procedures

A cross-sectional, web-based survey was sent to families who had a virtual Gender Clinic visit at BCCH during the first 6 months since the onset of the COVID-19 pandemic.

BCCH is the only tertiary children’s hospital in British Columbia, Canada. Since 1988, the Gender Clinic at BCCH has provided endocrine care for trans youth across the province. In the year prior to the COVID-19 pandemic, the Gender Clinic received approximately 200 new referrals, and more than 350 unique youth were seen for their initial or ongoing care. Most youth had 1 to 3 yearly in-person visits with a multidisciplinary team. Prior to the COVID-19 pandemic, only a very small number of virtual gender-related visits were attempted by the Gender Clinic. On 16 March 2020, all scheduled non-urgent in-person health visits were canceled in line with provincial health orders amidst the COVID-19 pandemic. Trans youth and their families had the opportunity to continue their routine care by connecting with their physician on a virtual visit using Zoom or Skype for Business, and virtual visits were also offered to new referrals. Youth and their caregivers received information and instructions by email and telephone from clinic administrative staff in preparation for the virtual visit. Virtual platforms used were available at no cost to families but required an internet-connected device. In-person appointments were arranged after an initial visit when a physical exam was deemed urgent or necessary for deciding ongoing management.

The survey was launched online in September 2020. An email was sent to potential participants introducing the study and outlining risks and benefits. Those who consented to participate were directed to an online survey link. A total of 2 survey completion reminders were emailed over 2 weeks.

The survey was designed by an interdisciplinary team consisting of pediatric endocrinologists, nurse clinicians, a social worker, and endocrinology trainees. It was pre-tested with trans youth (*n* = 5) and family members (*n* = 5) from the Gender Clinic, who were asked to review the survey for comprehension, ease of completion, relevance of response options and language and were encouraged to provide their feedback. Comments from the group that pre-tested the survey resulted in minor modifications to survey language and the change in response options for questions on differences between in-person and virtual visits.

This was a quality improvement study approved by the Research Privacy Advisor (Provincial Health Services Authority), as was required at our institution. Consent from participants was obtained at the time of the survey administration, and participants were informed that they could withdraw their consent at any time. Data were collected in accordance with the agency’s privacy laws. The proposal meets A pRoject Ethics Community Consensus Initiative (ARECCI) Ethics Screening Tool criteria for Quality Improvement and Evaluation projects [30,31]. As the main purpose of our study was the quality improvement and monitoring, it did not fall under the scope of the Research Ethics Board as per the University of British Columbia Guidance notes, Article 4.4.1, and Tri-Council Policy Statement 2 Article 2.5 [32].

Research Electronic Data Capture (REDCap) was used to build and administer the online survey and store survey data. REDCap is a secure, web-based application designed to support data capture for quality improvement/quality assurance and research studies.

### 2.2. Participants

Inclusion criteria were: (i) being a trans youth or parent/caregiver of trans youth (ii) having a virtual Gender Clinic visit at BCCH between 16 March 2020, and through until 31 August 2020; (iii) being able to understand and communicate in English. For trans youth completing the survey, assistance from an adult was required if they were less than 13 years old and optional (as-needed) for older youth.

### 2.3. Measures

#### 2.3.1. Socio-Demographic and Clinical Characteristics

Demographic and basic clinical information from the youth, including age at visit, sex assigned at birth, gender identity, current endocrine medications, duration of follow-up at BCCH and distance from the center were obtained from the medical record.

#### 2.3.2. Telehealth Usability Questionnaire

The survey included all questions from the Telehealth Usability Questionnaire (TUQ) [33]. Usability was defined as the extent to which a product can be used by specific users to achieve specific goals in a specific context of use [34]. The TUQ is a tool designed to evaluate the quality of newer-generation virtual care platforms, including usefulness, ease of use, reliability, interface and interaction quality, and satisfaction. This questionnaire incorporates items from rigorously validated measures in telehealth and has strong content validity. It has been assessed using various telecommunication systems to ensure its operational integrity and quality. It has been shown to have very good performance in regard to internal consistency, with Cronbach’s coefficient alpha values for all domains ranging between 0.81 and 0.93 [33]. All 21 questions from the TUQ were included in our survey, with no questions altered in intention or meaning. Questions were answered using a 4-point Likert scale, with response categories of “Not at all/Partly/Quite a bit/Completely”.

#### 2.3.3. Differences between Virtual and In-Person Visits, Challenges, and Future Preferences

The survey also included additional questions developed to explore differences between in-person and virtual gender visits. Participants were asked to compare the safety, stress, and comfort of virtual and in-person visits. A 3-point Likert scale (“virtual visits were better”/“both types of visits were the same”/“in-person visits were better”) was used for these questions. Participants were asked to report challenges in the use of virtual care by choosing one or more options from a provided list as well as writing free text. They were also asked about future visit preferences, including their desired frequency of in-person and virtual appointments post-pandemic.

### 2.4. Data Analysis

Data were analyzed via descriptive and inferential statistics. Likert scale results were presented in both percentages and frequencies as well as mean and standard deviation (SD). Differences in outcomes between caregiver and youth answers were analyzed using Welch’s unequal variances t-test. The use of t-tests is justified as assumptions of normality of the sample mean have been shown to be robust with Likert scales [35].

We performed an ordinal logistic regression analysis to understand the relationship between the outcome of the desire to continue virtual care after COVID-19 and patient characteristics: age at visit (years), sex assigned at birth (male/female), current endocrine medications (puberty blockers only/hormone therapy/no medications), starting a medication after the clinic visit (yes/no), duration of follow-up at the BCCH Gender Clinic (months), first clinic visit being virtual (yes/no), distance from the center (50-km intervals), and who completed the survey (trans youth/caregiver). Based on our initial results, we also performed a binary logistic regression analysis to explore associations between the perceived barrier of “it is hard to build a relationship with the provider over a virtual visit” and select patient characteristics (age, sex assigned at birth, duration of follow-up, first clinic being virtual) as well as who completed the survey. Unadjusted and adjusted odds ratios and 95% confidence intervals (CI) were estimated based on multivariable logistic regressions. Nagelkerke pseudo-R^2^ was reported as a measure of effect size for each model [36].

Statistical significance was set at *p* < 0.05. Microsoft Excel 2020™, R version 4.0.2, and SPSS Version 27 were used for analyses.

## 3. Results

The survey was emailed to 221 families. A total of 94 initiated the survey; 5 did not subsequently consent, and 2 did not provide responses and thus were excluded. Hence, 87 responses were included in the analysis, resulting in a 39% response rate. Most surveys were completed by parents, family members, or guardians (*n* = 59), who will be referred to as “caregivers,” while trans youth themselves were involved in completing one-third of the surveys (*n* = 28, 18 of them alone, and 10 together with a family member).

Table 1 summarizes the characteristics of trans youth whose visits were evaluated. Trans youth who completed the surveys were older than those whose surveys were completed by caregivers, and they were receiving different endocrine treatments; no other significant differences were found among these two groups. 15% of participants were having their first Gender Clinic visit. A total of 20% of participants started a new medication after the virtual encounter. Both caregivers and trans youth participated together in 75% of the virtual clinic visits, 22% of visits involved the trans youth alone, and only 3 virtual visits occurred with just the caregiver alone. All visits included a staff endocrinologist, while a doctor-in-training and a nurse took part in 23% and 10% of visits, respectively, and one visit included a social worker.

In terms of distance to the Gender Clinic and travel time, 45% of families lived within 50 km from the clinic and 39% between 50 and 100 km, while 5% lived more than 500 km away. A total of 30% of participants reported that virtual visits would save two or more hours of one-way travel. There were 48% of caregivers and 40% of trans youth that would miss between 4 and 10 h of school and/or work to come to an in-person visit, and 10% and 14%, respectively, that would miss more than 10 h. As for the cost to attend an in-person visit, 62% of families would spend less than 50 Canadian dollars, while 7% and 8% would need to spend between 200–500 and more than 500 dollars, respectively.

**Figure 1:**
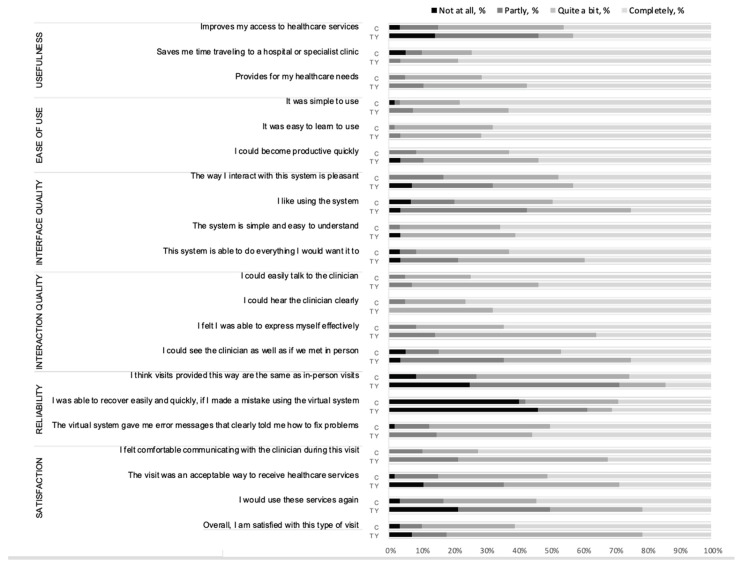
Usability of virtual gender visits for caregivers and trans youth: distribution of responses to the Telehealth Usability Questionnaire. C = caregivers; TY = trans youth.

Table 2 and Figure 1 illustrate the responses of caregivers and trans youth regarding the usability of virtual care visits. Mean scores for each of the 6 categories of the Telehealth Usability Questionnaire ranged between “quite a bit” and “completely.” The highest scores were given to usefulness, ease of use and interaction quality. All survey respondents thought that virtual visits met their healthcare needs at least “partly.” Virtual visits were perceived as an acceptable way to deliver healthcare at least “partly” by 98% of caregivers and 90% of trans youth. Trans youth reported lower usability scores compared to their caregivers; this was statistically significant for the cumulative overall usability score and many individual questions, including all items under the “satisfaction” category. Despite these differences, mean usability scores provided by youth also ranged between “quite a bit” and “completely” for all items in the TUQ, except for two that were rated as “partly”: virtual visits being the same as in-person visits and the system providing clear error messages; the latter question was rated as “partly” by caregivers too.

**Figure 2:**
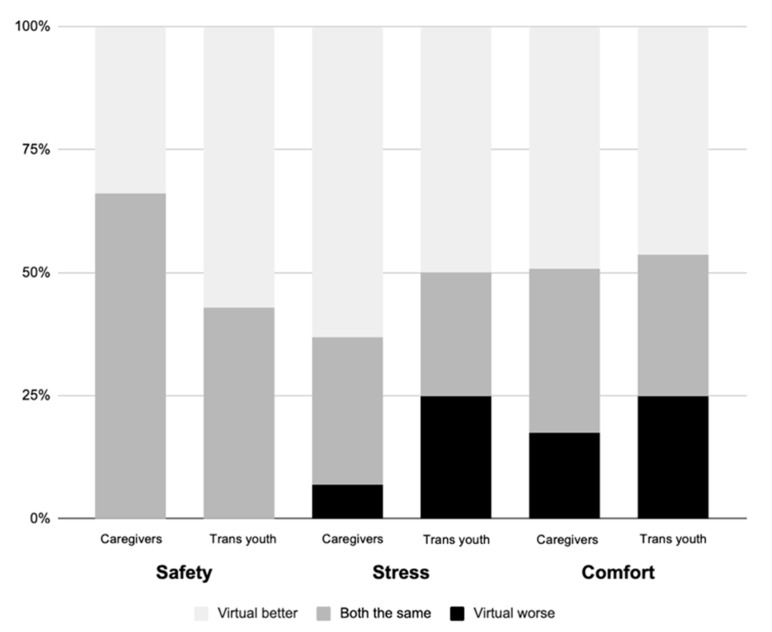
Safety, stress, and comfort of virtual visits: perceptions of caregivers and trans youth.

Figure 2 shows participant perspectives regarding safety, stress, and comfort of virtual visits, compared to in-person visits. All families described virtual visits as equally safe or safer than in-person, and none reported that virtual visits were less safe. The majority considered virtual visits to be equivalent or better than in-person visits in terms of comfort and stress. There were no statistically significant differences between caregivers and trans youth responding to these questions. However, there was a trend towards trans youth being more likely to find virtual visits safer than in-person, compared to their caregivers (59% vs. 33%, *p* = 0.07).

Regarding barriers to virtual visits, 69% of participants reported that “it was not difficult” to have a virtual visit, but 29% stated that it was “hard to build a relationship” with healthcare providers on a virtual appointment, and 13% had “trouble focusing during the virtual visit”. Among other issues, 8% were “not familiar with the technology”, 7% had “problems accessing a computer or internet”, and the same proportion did not have “a quiet and private place”. A small number of families (2%) reported that “the technology did not feel safe and secure”. No other barriers were reported by participants. On multivariable logistic regression analysis (Table 3), the age of trans youth being part of the visit was inversely associated with difficulty building a relationship with their physician. There was also a trend towards trans youth who completed the surveys themselves being more likely to report difficulty establishing a relationship over a virtual visit compared to caregiver responses. The Nagelkerke pseudo R^2^ indicates that 9% of the variation in results for this question may be accounted for by the model.

When considering their future care, 94% of participants stated that they would like to have virtual visits continued following the COVID-19 pandemic. On multivariable logistic regression analysis, no significant associations were found regarding the desire for ongoing virtual care and relevant participant characteristics, including no association with whether a caregiver or youth provided the assessment. Families who were new to the Gender Clinic appeared less likely to want to continue virtual care in the future, however, this trend was not statistically significant. The Nagelkerke pseudo R^2^ value for this model was 18%. The majority of participants (85%) would choose a combination of both virtual and in-person visits in the future, while 9% would prefer only virtual care, and 6% would prefer all in-person care. The mean number of yearly visits desired by trans youth and caregivers was three in total, consisting of one in-person and two virtual visits per year. Many participants would like a nurse or social worker from the Gender Clinic team (55% and 38%, respectively) and other community providers (18%) to be present, in addition to the Gender Clinic physician. A total of 77% of participants would like to have various members of the Gender Clinic team join in the same visit instead of having separate visits with each.

## 4. Discussion

Our study reflects the perspectives of trans youth and their caregivers on virtual care. To our knowledge, this is the first study to assess the usability of gender-related virtual visits using a validated tool specifically developed for the evaluation of newer telemedicine platforms. The results show a very positive assessment of virtual care by trans youth and their families.

With the postponement of in-person non-urgent appointments, virtual care has become part of routine care, not only in our hospital but also in many centers worldwide. In this setting, the family-reported usability of these virtual visits was positive in all areas, especially usefulness, ease of use, and interaction quality. An overwhelming majority of participants reported that it was easy to talk to their clinician during their virtual visits and they could express themselves effectively. This is particularly notable as the study occurred during the initial stages of the pandemic, when both physicians and families were still early on in adapting to this new way of providing and receiving health care. In line with the rapid nature of the transition, it was not unexpected to find that reliability had the lowest scores.

A study assessing the attitudes of trans youth (*n* = 12) and their caregivers (*n* = 27) receiving virtual gender-affirming care during the COVID-19 pandemic reported virtual visits to be the same or better than in-person visits [28]. Another recent study evaluating the experience of trans youth who had had a virtual gender visit (*n* = 57) found that most of them were satisfied and interested in having telemedicine as an option for ongoing care [29]. Our study now adds to this knowledge, supporting the robustness of gender virtual visits by presenting youth and caregiver assessments of usability of virtual visits in this setting.

Transgender people have been shown to have worse general and mental health outcomes than their cisgender counterparts. The reasons for this include stigma, discrimination, minority stress, and socioeconomic differences, among others [37,38,39,40]. The current global pandemic has accentuated health disparities among trans youth and other marginalized identities [18,19]. In this difficult context, virtual care has ensured the ongoing provision of gender-affirming care for this population. Furthermore, we found that the care provided by our gender clinic by virtual visits was rated highly in regard to usability, and importantly, all trans youth and families in our study considered that virtual visits provided for their healthcare needs. Given the very well-known benefits of gender-affirming care [8,9,10,11], these results should be interpreted as a call to action to continue making efforts to avoid withholding or delaying care due to barriers to in-person visits.

It is clear that trans youth are a vulnerable population. According to a recent Canadian survey, two-thirds had experienced discrimination because of their gender identity, and a significant proportion suffered sexual harassment or physical violence [16]. Health services do not appear to be an exception: in different studies, this group highlighted significant challenges, concerns, and negative experiences in health care settings [41,42]. Considering this, ensuring that trans youth feel safe during their gender care visits should undoubtedly be a priority, and virtual delivery of care may be advantageous in this aspect. We found that trans youth and families considered virtual visits to be as safe or safer compared to in-person visits, and no one perceived virtual visits to be less safe. Supporting our results, many trans youth in the study by Sequeira et al. reported feeling safer and less anxious with virtual visits [29]. In addition, families in the study by Apple et al. reported feeling comfortable with virtual visits and described them as the same or better than in-person visits for privacy and provider communication [28]. While reasons for this should continue to be explored, the opportunity to provide gender-affirming care that is perceived as safe by this group should not be disregarded, even when it becomes possible to return to in-person care.

Further, with regards to supporting a vulnerable patient population, virtual care has the potential to overcome access barriers resulting from the limited number of multidisciplinary gender clinics and their locations in urban centers [39]. In a survey of over 1500 trans youth across Canada, transportation and cost were mentioned among the reasons for not getting adequate care by almost 30% of the group [16]. In our study, trans youth and families revealed the multiple ways in which virtual care provides an easier, less time-consuming, and less expensive option to receive care. A substantial proportion of our participants lived close to the hospital, which could reflect location-based disparities in access to gender-related care, but even so, we have learned that a trip to our center can mean significant disruption in terms of time, travel and cost for some families. Most trans youth and caregivers would miss a half or full day of work or study to attend in-person visits. In addition, despite provincial health insurance covering the fees for medical services, many families spend considerable amounts of money to come to in-person visits. Addressing cost and distance barriers faced by a marginalized group should continue to be a valuable advantage of virtual gender-affirming care, even in a post-pandemic setting.

In a survey performed before the onset of the COVID-19 pandemic, trans youth reported that they were interested in telehealth-supported gender-affirming care [18]. Taking a step forward, here we find now that trans youth and their caregivers who had virtual visits during the early stages of the COVID-19 pandemic are satisfied with these visits and are hopeful to continue receiving virtual care. Only a very small fraction of our participants would like to return to in-person visits for all of their gender-affirming care after the pandemic, and 94% request virtual care to be a part of their future. In previous studies, preferences and attitudes toward virtual care for trans youth varied based on the specific reason for the visit; for instance, telemedicine was less likely to be chosen over in-person appointments to prepare for surgery or learn to give hormone self-injections [22,28]. This would further support both the need for a combination of in-person and virtual appointments, which according to our results, is by far the most desired model of care, and also the importance of tailoring care provided to each person’s context and demands.

Given the unique needs of trans youth, there is general agreement that their care requires a multidisciplinary approach [4]. Not surprisingly, most families would like all members of their healthcare team (specifically a Gender Clinic nurse and social worker) to be involved in coordinated multidisciplinary visits in the future. This is a key finding that will lead to a review of our clinic workflow to ensure the provision of gender care in keeping with youth and family expectations.

When the COVID-19 pandemic emerged, the shift from in-person to virtual care was fast and not optional. In contrast, returning to in-person visits after this pandemic can be stepwise and carefully designed. Given the advantageous aspects of virtual care and its remarkable usability, it is highly likely that a proportion of patients will benefit from continuing this model of care. To provide equitable and family-centered gender care, we must seek to identify youth and families that will gain the most from, or alternatively, that may experience distress from using virtual healthcare. Families who had a virtual appointment for their first Gender Clinic visit appeared to be less likely to wish to continue virtual care in the future, compared to families who had previously had in-person visits. In addition, nearly one-third of participants in our survey stated that they found it hard to build a relationship with their healthcare providers over a virtual visit; younger age of trans youth was associated with more difficulty in this regard, and there was a trend towards trans youth themselves finding it harder to build a relationship with their physician than caregivers. The apparent modest effect size on multivariable logistic analysis reflects the need to continue to uncover patient and family characteristics that are associated with these outcomes. Our results highlight areas for more focus as we work to optimize the delivery of care in the context of the evolving pandemic and beyond.

While the majority of our participants reported a positive experience with virtual visits and would like to continue virtual gender-related care in the future, trans youth showed lower usability rates than caregivers. Prior studies have shown similar findings and have hinted at some reasons why trans youth may have more difficulty with virtual care. Apple et al. found that trans youth were significantly less likely than caregivers to report that telehealth was the same or better than in-person visits for addressing their concerns [28]. In the study by Sequeira et al., some trans youth had requested to disable their camera or the self-view capacity during visits, which the authors attribute to dysphoria related to seeing their image on their screen. In addition, in that study, which included only trans youth, participants expressed that they would prefer in-person visits, despite their positive views on virtual gender-related care [29]. Lack of a safe and confidential place for a virtual visit has also been previously described as a barrier in this population [39]; however, this was identified as a concern by only a small proportion of participants in our study. Further research centered on understanding the healthcare experience of trans youth will be of key importance to continue improving virtual care for this group.

A strength of this study is that it reflects the perspectives of youth and caregivers from one of North America’s largest pediatric gender clinics. This study has a number of limitations. First, surveys were performed in the early months of the COVID-19 pandemic, and it is likely that youth and caregiver experience and preferences will continue to evolve. In addition, we had a modest response rate. This appears to be a relatively frequent issue for online surveys, and it is possible that it might have been exacerbated by the pandemic [43,44]. While it could be postulated that the characteristics of this population may have also contributed to the low response rate, current studies evaluating survey responses among sexual minorities do not support this assumption [45]. Similar studies evaluating virtual gender visits for trans youth had response rates as low as 15% and up to 56%; interestingly, while recruitment in the first was conducted via text message, email, or messages through electronic medical records, participants in the latter study were also contacted by phone [28,29]. Moreover, it is possible that participants who replied to this survey could be more likely to engage in virtual visits, which could constitute selection bias. Another limitation is that a significant portion of surveys was completed by family members who participated in the visit, as opposed to trans youth themselves. In addition, as younger participants completed the surveys with the assistance of an adult, caution should be exerted when evaluating differences between youth and caregivers. Alternative recruitment strategies, such as contacting youth directly rather than using provided email addresses (mainly directed to caregivers), might help improve the response by trans youth in the future. Further studies should aim to gather the perspectives of younger youth by different methods, including interviews or survey questions specifically developed for this group, to ensure that their answers reflect their own opinion. Finally, there are aspects that are particularly relevant to adolescents that are not included in the TUQ and have been mentioned as challenges to gender virtual visits in previous studies, such as privacy [28]. While our survey included additional questions, including barriers to virtual care, further qualitative studies with a focus on the perspectives of youth are needed. Follow-up interviews with healthcare professionals who have been part of these visits may also add great value.

## 5. Conclusions

We describe the usability of virtual gender-related visits for trans youth at our Gender Clinic during the COVID-19 pandemic. During this time of unprecedented change, this study revealed an extraordinarily positive response from trans youth and their caregivers, who were satisfied with virtual care. All participants considered this type of visit as safe or safer than in-person appointments. In addition, virtual care was reported to be beneficial in terms of cost and travel time. In a shift from our pre-pandemic provision of care, families desire a combination of virtual and in-person visits moving forward. While these results are certainly encouraging, continuous assessment and improvement will be essential to ensure the provision of high-quality care. With the valuable information gathered in this study, our clinic’s delivery of care will continue to be transformed in an effort to achieve optimal patient- and family-centered care.

## Figures and Tables

**Table 1:** Characteristics of trans youth participating in virtual gender visits.

	All Participants *n* = 87	Caregiver Filled the Survey *n* = 59	Youth Filled the Survey *n* = 28
Age (years), mean (SD)	15.55 (3.01)	14.63 (2.65)	17.48 (2.84)
Duration of follow-up at Gender Clinic (months), mean (SD)	21.2 (20.5)	19.4 (21.3)	25 (18.5)
Sex assigned at birth, female, n (%)	52 (60)	33 (55)	19 (67)
Reported gender identity (%)			
Trans male/trans masculine	46 (53)	28 (47)	18 (64)
Trans female/trans feminine	35 (41)	26 (44)	9 (32)
Gender non-binary	3 (3)	3 (5)	0
Other	3 (3)	2 (3)	1 (4)
Current endocrine therapies, n (%)			
No medications	21 (23)	19 (32)	2 (7)
Leuprolide	31 (35)	26 (44)	5 (17)
Testosterone	29 (33)	11 (19)	18 (64)
Estrogen	13 (15)	8 (14)	5 (18)
Spironolactone	5 (6)	1 (2)	4 (14)
Bleeding cessation and other	4 (5)	3 (5)	1 (4)

**Table 2:** Usability of virtual visits: Telehealth Usability Questionnaire responses by caregivers and trans youth ^1^.

	Caregiver Filled the Survey *n* = 59			Youth Filled the Survey *n* = 28			*p*-Value
	Mean	SD	Range	Mean	SD	Range	
**Usefulness**	3.5	0.5	1–4	3.4	0.6	1–4	0.12
A virtual visit improves my access to healthcare services	3.3	0.8	1–4	2.8	1.2	1–4	0.04 *
A virtual visit saves me time traveling to a hospital or specialist clinic	3.7	0.7	1–4	3.8	0.5	2–4	0.9
A virtual visit provides for my healthcare needs	3.7	0.6	2–4	3.5	0.7	2–4	0.18
**Ease of Use and Learnability**	3.6	0.5	1–4	3.5	0.6	1–4	0.34
It was simple to use the virtual system	3.7	0.6	1–4	3.5	0.7	2–4	0.12
It was easy to learn to use the virtual system	3.7	0.5	2–4	3.7	0.6	2–4	0.96
I could become productive quickly using the virtual system	3.5	0.7	2–4	3.4	0.8	1–4	0.31
**Interface Quality**	3.4	0.6	1–4	3.1	0.6	1–4	0.05
The way I interact with the virtual system is pleasant	3.3	0.8	1–4	3.0	1.0	1–4	0.16
I like using the virtual system	3.2	0.9	1–4	2.8	0.9	1–4	0.02 *
The virtual system is simple and easy to understand	3.6	0.6	2–4	3.5	0.7	1–4	0.47
This virtual system is able to do everything I would want it to do	3.5	0.8	1–4	3.1	0.7	1–4	0.05
**Interaction Quality**	3.6	0.6	1–4	3.3	0.5	1–4	0.04 *
I could easily talk to the clinician using the virtual system	3.7	0.6	2–4	3.4	0.6	2–4	0.1
I could hear the clinician clearly using the virtual system	3.7	0.6	2–4	3.7	0.5	3–4	0.63
I felt I was able to express myself effectively using the virtual system	3.6	0.7	2–4	3.2	0.7	2–4	0.02 *
I could see the clinician as well as if we met in person	3.3	0.9	1–4	2.9	0.9	1–4	0.02 *
**Reliability**	2.9	0.8	1–4	2.6	0.8	1–4	0.07
Visits provided this way are the same as in-person visits	2.9	0.9	1–4	2.2	1.0	1–4	0.0006 *
I was able to recover easily and quickly, if I made a mistake using the virtual system	3.4	0.8	1–4	3.4	0.8	2–4	0.37
The virtual system gave me error messages that clearly told me how to fix problems	2.5	1.3	1–4	2.2	1.3	2–4	0.96
**Satisfaction and Future Use**	3.5	0.5	1–4	2.9	0.8	1–4	0.007 *
I felt comfortable communicating with the clinician during the virtual visit	3.6	0.7	2–4	3.1	0.7	2–4	0.001 *
The virtual visit was an acceptable way to receive healthcare services	3.3	0.8	1–4	2.8	1.0	1–4	0.01 *
I would use these services again	3.5	0.8	1–4	2.5	1.1	1–4	0.0004 *
Overall, I am satisfied with this type of visit	3.4	0.5	1–4	2.9	0.8	1–4	0.005 *
**Overall usability**	3.4	0.8	1–4	3.1	0.9	1–4	0.01 *

^1^ Likert scale answers: 1 = not at all, 2 = partly, 3 = quite a bit, 4 = completely; * *p* < 0.05.

**Table 3:** Association between patient characteristics and difficulty building a relationship with their provider on a virtual visit and desire to continue virtual care: multivariable regression analysis.

	Perceived Hard to Build a Relationship with Provider ^1^			Would Desire to Continue Virtual Visits ^2^		
	aOR	95% CI	*p*-Value	aOR	95% CI	*p*-Value
Age (years)	0.81	0.67–0.99	0.04 *	1.06	0.73–1.53	0.76
Sex assigned at birth (male)	0.82	0.28–2.38	0.71	0.87	0.19–3.98	0.86
Duration of follow up (months)	1	0.98–1.03	0.89	0.99	0.96–1.03	0.71
Distance from hospital (50-km)	-		-	0.95	0.8–1.13	0.56
First visit was virtual (yes)	0.63	0.12–3.27	0.56	0.06	0–1.15	0.06
Current medications	-		-			
- puberty blockers				0.54	0.03–10.15	0.678
- hormone therapy				1	0.03–36.24	0.998
Starting a medication after virtual visit	-		-			
- puberty blockers				11.84	0.7–199.8	0.087
- testosterone				4.83	0.2–119.58	0.336
- estrogen				6.29	0.38–104.07	0.199
Trans youth filled out the survey (yes)	2.79	0.85–9.16	0.09	1.25	0.26–6.01	0.78

* *p* < 0.05 is 0.18, ^1^ pseudo R^2^ = 0.09, ^2^ pseudo R^2^ = 0.18.

## Data Availability

Due to the nature of this study, participants of this study did not agree for their data to be shared publicly, so supporting data is not available.
